# Correlation between Plasma DNA and Tumor Status in an Animal Model

**DOI:** 10.1371/journal.pone.0111881

**Published:** 2014-12-02

**Authors:** Naoko Sueoka-Aragane, Akemi Sato, Naomi Kobayashi, Masaru Ide, Masako Yokoo, Yumi Nagano, Eisaburo Sueoka, Seiji Okada, Shinya Kimura

**Affiliations:** 1 Division of Hematology, Respiratory Medicine and Oncology, Department of Internal Medicine, Faculty of Medicine, Saga University, Saga, Japan; 2 Department of Laboratory Medicine, Saga University Hospital, Saga, Japan; 3 Division of Hematopoiesis, Center for AIDS Research, Kumamoto University, Kumamoto, Japan; University of Nebraska Medical Center, United States of America

## Abstract

Overcoming metastasis is one of the most important issues with lung cancer. Since metastasis arises through complex steps, a suitable animal model is indispensable for investigation of metastasis. To establish an animal model reflecting human metastatic lung cancers, we used NOD/SCID/Jak3^null^ (NOJ) mice, which exhibit deficiencies in NK cell activity, macrophage and dendritic cell function, and complement activation, as well as T and B cell deficiencies. After screening twenty human lung cancer cell lines through expression patterns of E-cadherin and vimentin according to epithelial mesenchymal transition features, an H1975 cell line carrying *EGFR* mutations, L858R and T790M, was selected for investigation. Inoculation of the cells into the dorsal flanks caused systemic metastases after one month in lymph nodes, liver, lung, and peritoneum, suggesting that metastases occurred both lymphogenically and hematogenously. We confirmed the existence of H1975 cells in metastatic lesions by detection of T790M and L858R using the mutation-biased PCR and quenching probe (MBP-QP) system previously established in our laboratory. In addition, tumor-derived plasma DNA could be detected using the MBP-QP method. The amount of tumor-derived DNA was associated with tumor volume, whereas an unrelated large amount of tumor-derived DNA was circulating in the presence of metastasis. We present a novel animal model with systemic metastasis with human lung cancer cells. The amount of tumor derived DNA would be related with tumor volume and tumor progression such as metastasis.

## Introduction

Lung cancer is the leading cause among deaths due to malignant tumors. One factor leading to difficulty in treating lung cancer is a high frequency of metastases. Lymphogenic and distant metastases were found in 46% and 21% of diagnoses, respectively, and most recurrence after surgery occurs as distant metastasis [1], [2]. Improving the prognosis of lung cancer patients cannot be expected without overcoming metastasis.

It has been suggested that metastases may arise by evolution of cancer cells during treatment [3], [4]. In the primary lesion, heterogeneity has been evidenced in terms of both molecular alterations and morphological aspects. Among the cancer cells in the primary lesion, those that acquire metastatic potency spread throughout the body, with the result that molecular characteristics and sensitivity to anti-cancer agents sometimes differ between primary and metastatic lesions [5]–[8]. In addition, metastasis consists of several steps such as loss of cell adhesion, intravasation, survival in circulation, exit into new tissues, and colonization in a distant site [9], [10]. Therefore, a suitable animal model would be indispensable for investigation of metastasis and screening potential anti-cancer agents targeted at metastasis.

Animal models for studying metastasis consist mainly of two types: genetically engineered models of cancers and transplantable tumor model systems [11], [12]. The advantage of genetically engineered models is that we can observe the process of carcinogenesis from early to advanced stages. Considering the goal of establishing metastatic models reflecting human cancers, it is better to use human cancer cells. Therefore, we chose the xenograft model of human lung cancer cells using an immunodeficient mouse. The immunodeficient mouse model, such as the severe combined immunodeficient (SCID) mouse, which is defective in T and B cell development, and the non-obese diabetic SCID (NOD/SCID) mouse, which additionally has defects in NK cells, macrophage function, and circulating complement, have been used for transplantation of hematological malignancies [13], [14]. Although these immunodeficient mice also have been used to investigate solid tumors, it is difficult to induce systemic metastasis. Recently, NOD/SCID interleukin-2 receptor gamma chain null (NOD/SCID *IL2rγ^null^*) mice had higher tumorigenic potential of melanoma by several orders of magnitude compared to NOD/SCID, suggesting that the common γ-chain of cytokine receptors may play a crucial role in tumor development [15]. To establish an animal model reflecting human metastatic lung cancers, we used NOD/SCID/JAK3^null^ (NOJ) mice produced by crossing NOD/SCID and JAK3^null^ mice [16]. JAK3 is a tyrosine kinase involved in signaling from the common γ-chain of cytokine receptors such as IL-2, 4, 7, 9, and 15, which contribute to proliferation and differentiation of lymphocytes [17]. Therefore, NOJ mice exhibit deficiencies in NK cell activity, macrophage and dendritic cell function, and complement activation, as well as T and B cell deficiencies. Using these mice, we established a highly metastasized animal model using a human lung cancer xenograft.

Recently, molecular analysis using plasma DNA has been investigated, since it is non-invasive and can be repeatedly monitored [18]–[22]. We established a system for monitoring *EGFR* mutation, the T790M using mutation-biased PCR and quenched probe (MBP-QP) method [18]. T790M is a gatekeeper mutation of *EGFR*, which appears in half of lung cancer patients treated with EGFR-TKI [23]–[25]. Because the system is highly sensitive, it was able to successfully detect T790M using plasma DNA. We therefore applied this technique to monitor metastasis in the animal model. Besides *EGFR* mutations, plasma DNA has been widely applied to detecting molecular alterations of cancers [20]–[22], [26]. However, the mechanisms regarding how tumor derived DNA is released into peripheral blood have not been clarified. In this paper, we present the animal model for metastasis with a non-invasive monitoring system using plasma DNA. The animal model could be applicable to screening for novel anti-cancer agents, especially those targeted to T790M. In addition, we show the relationship between tumor volume and amount of *EGFR* mutations, and discuss mechanisms of release of tumor derived DNA into peripheral blood.

## Materials and Methods

### Cell preparation and culture conditions

Twenty non-small cell lung cancer cell lines were purchased from the American Type Culture Collection (Manassas, VA). Cells were cultured in RPMI-1640 supplemented with 10% fetal bovine serum at 37^ο^C in 5% CO_2_. H1975 cells were cultured in RPMI-1640 containing 10 mM HEPES, 1 mM sodium pyruvate, 2.5 g/L glucose, and 1.5 g/L sodium bicarbonate, supplemented with 10% fetal bovine serum.

### Western blotting

Western blotting was performed using whole-cell lysates prepared from lung cancer cell lines using lysis buffer containing 50 mM Tris-HCL at pH 8.0, 150 mM NaCl, 5 mM MgCl_2_, 1% TritonX-100, 0.1% sodium dodecyl sulfate, 0.5% sodium deoxycholate, 40 mM sodium fluoride, 1 mM sodium orthovanadate, 1 µg/ml leupeptin, 10 µg/ml aprotinin, and 1 mM phenolmethylsulfonyl fluoride, as described previously [27]. Antibodies for E-cadheirn, vimentin, and actin were obtained from Santa Cruz Biotechnology, Inc. ImageJ software (NIH, Bethesda, Maryland) was used for the analysis.

### Animal experiment

NOD/SCID/JAK3^null^ (NOJ) mice were established as described previously [16]. They were housed under pathogen-free conditions in animal facilities at Saga University according to institutional guidelines. The protocol was approved by the Committee on the Ethics of Animal Experiments of the Saga University (Permit Number: 24-037-0). All surgery was performed under sodium pentobarbital anesthesia, and all efforts were made to minimize suffering. H1975 cells (1×10^7^) were injected into the dorsal flanks of NOJ mice. Tumor volumes, defined as ((short axis)^2^× (long axis))/2, and body weight were measured every 3 days. Mice were evaluated daily for signs of morbidity or tumor growth. When body weight was reduced by 10% of original weight, or metastatic manifestations such as superficial lymph node swelling or ascites were observed, the mice were euthanized with ether and dissected. All soft tissues were divided into 3 parts: two parts were frozen in liquid nitrogen for storage for evaluation of molecular alterations and protein analysis, and one part was fixed in 10% formaldehyde and processed for pathological examinations.

### DNA extraction from tissues or plasma

Genomic DNA was isolated from specimens using QIAamp DNA mini kits (QIAGEN, Hilden, Germany) according to the manufacturer's instructions. Peripheral blood samples from mice were collected into tubes containing sodium heparin. Plasma was immediately separated from blood cells by 3000 rpm centrifugation at 4°C for 20 min. Supernatants were collected and stored at -80°C until assays could be performed. DNA was isolated from 200 µl of patient plasma using a QIAamp DNA mini kit. DNA concentration was calculated based on absorbance at 260 nm.

### Mutation analysis by the MBP-QP method, and direct sequencing

*KRAS* codon 12/13 mutations were determined by a quenching probe system (QP), and *EGFR* T790M and L858R mutations were detected by the MBP-QP method, a modified QP-system using i-densy™ IS-5320 (ARKRAY Inc., Kyoto, Japan), as described previously [18], [19], [28]. Briefly, MBP-QP is a fully automated system with two steps: mutation-biased PCR (MBP) and QP-system analysis. MBP was designed to increase efficiency of amplification of mutants, which are determined by monitoring the fluorescence intensity of a TAMRA-conjugated, guanine-specific quench fluorophore probe (QProbe, J-Bio21, Tokyo, Japan). QProbes are complementary to mutants in T790M and L858R detection systems, and to wild type in KRAS codon 12/13 mutation detection system. The analyses were performed using i-densy (ARKRAY Incorporated, Japan). The areas under the mutation peaks were determined by the “idensy AreaAna” software, developed by ARKRAY Incorporated. The other mutation analyses were performed by direct sequencing using primer sets shown in Table S1.

### Statistical analysis

The correlation between area under the mutation peaks of T790M or L858R and total amount of genomic DNA isolated from H1975 was tested using Spearman's rank correlation for continuous variables. The correlations between area under the mutation peaks of T790M or L858R and tumor volumes were also examined using the same method. The correlation of area under the mutation peaks and status of metastasis was examined using the nonparametric Mann-Whitney U test for continuous variables. These analyses were performed using IBM SPSS Statistics 19 (SPSS Inc., IBM Company).

## Results

### Screening of non-small cell lung cancer cell lines for animal model of metastasis

**Figure 1:**
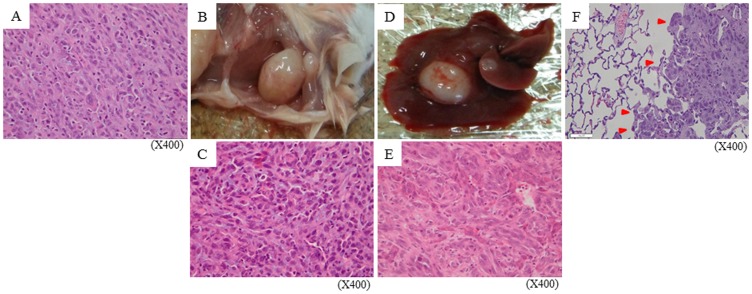
Metastatic lesions in lymph nodes, liver, and lung. Mouse no. 6 in Table 1 was dissected 110 days after inoculation because of axillary lymph node swelling. (A) HE staining of primary lesion. Macroscopic appearance (B) and HE staining (C) of metastasis into axillary lymph node. Macroscopic appearance (D) and HE staining (E) of metastasis into liver. (F) HE staining of lung metastatic lesion. Red arrowheads indicate metastasis.

The twenty human lung cancer cell lines were screened through pattern of E-cadherin and vimentin using Western blot analysis on the basis of definition of epithelial mesenchymal transition, which is a fibroblastoid phenotype, loss of E-cadherin, and gain of vimentin [29], [30]. We found seven cell lines with high expression of vimentin and negligible expression of E-cadherin (high vim/low E-cad), and eight cell lines with high expression of E-cadherin and negligible expression of vimentin (high E-cad/low vim) (Fig. S1). To compare these two groups in terms of occurrence of metastasis, H1975, H1792, and H226B as high vim/low E-cad, and PC-9 and H322 as high E-cad/low vim were used. 1×10^7^ cells/mouse of each cell line were injected into the dorsal flanks, and these mice were sacrificed when body weight was reduced by 10% of original body weight if the mice didn't die naturally. All mice inoculated with H1975 or H226B cells were engrafted with metastasis, whereas H1792 cells were not engrafted in any of the 5 mice (Fig. S2). H322 cells did not show metastasis over 100 days after inoculation although cells were engrafted in all seven mice which were examined (data not shown). All of the PC-9 inoculated mice evidenced 10% reduction of body weight from 27 to 63 days, and were dissected, but metastasis was not observed in any of these mice (data not shown). Further investigations were performed using H1975 cells because these cells carry *EGFR* mutations, L858R and T790M. Metastatic lesions were investigated using 15 mice injected with H1975 cells. One mouse out of the 15 died 35 days after inoculation from unknown etiology. Except for that mouse, metastatic profiling is shown in Table 1. Metastases into lymph nodes, liver, and lung, as well as peritoneal dissemination, occurred in 13 mice from 40 to 113 days (Table 1, Fig. 1). The frequency of metastasis to axillary and abdominal lymph nodes was 79% (11/14), and metastasis to lung and liver occurred in 64% (9/14) (Table 2). Peritoneal dissemination with ascites was also observed in 29% (4/14) of those mice. In total, one or more of these types of metastasis was observed in 93% (13/14) of the mice.

**Table 1:** Metastatic profiling of H1975 xenograft model.

No. of Mice	Dissection (days)	Size of primary lesions (cm3)*	Dissemination	Metastatic lesions		
				Lymph nodes	Liver	Lung
1	91	10.1	-	+	-	+
2	91	13.5	-	+	-	-
3	91	15,6	+	-	-	+
4	91	6.1	-	-	-	-
5	73	5.6	+	+	-	-
6	110	15.6	-	+	+	+
7	110	18.5	-	+	+	+
8	110	11.9	-	+	-	+
9	113	6.3	-	+	-	+
10	113	6.6	-	+	-	+
11′	113	6.6	-	+	-	-
12	110	I2.6	-	+	-	+
13	40	0.9	+	+	-	-
14	106	15,7	+	-	-	+

*Size of primary lesions was calculated as ((short axis)^2^× (long axis))/2.

**Table 2:** Frequency of metastasis and/or dissemination in H1975 xenograft model.

	(n = 14)
Metastatic/disseminated lesions	Frequency
Metastasis to lymph nodes	79%(11/14)
Metastasis to organs	64% (9114)
Dissemination	29% (4/I 4)
Any ofthe above	93%(13/14)

### Analysis of somatic mutations of primary and metastatic lesions

**Figure 2:**
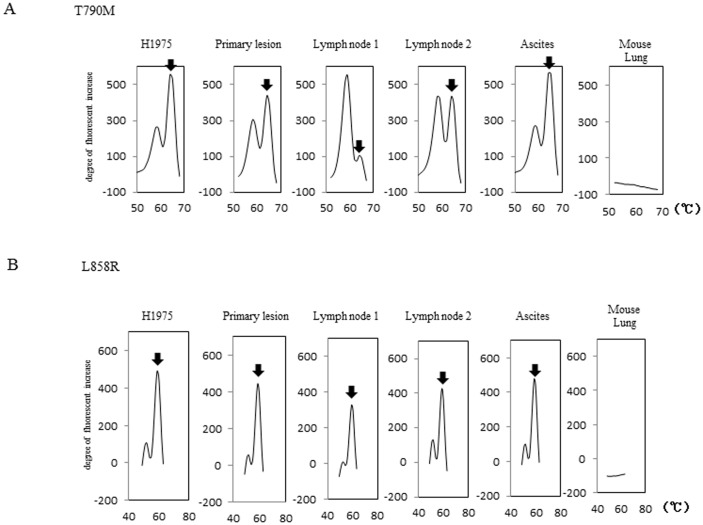
Detection of T790M and L858R mutations in both the primary lesion and metastatic lesions. Mouse no. 5 in Table 1 was dissected 73 days after inoculation because of the presence of ascites. Genomic DNA was isolated from primary and metastatic lesions such as lymph nodes and ascites. The MBP-QP method was applied to 4 µl of purified DNA as described in “Materials and methods” for detection of T790M (A) and L858R (B). Genomic DNA isolated from normal mouse lung and H1975 were used as negative and positive controls, respectively. Black arrows indicate the peaks for T790M or L858R mutations.

Homologies of *EGFR* between human and mouse at exons 20 and 21 were 88.2% and 89.7%, respectively. To distinguish *EGFR* mutations between mouse and human, primer sets and probes for MBP-QP were designed as shown in Tables S2 and S3. These mutations were detected in all metastatic lesions as well as in primary lesions inoculated with H1975 cells, confirming that all metastatic lesions originated from subcutaneous tumors. Figure 2 shows representative results from mouse no. 5. This assay system did not work using genomic DNA isolated from mouse lungs. The areas under mutation peaks of T790M and L858R were correlated with the exception of lymph node 1. T790M was mainly located on the same DNA allele as L858R, but occasionally it was located in trans (data not shown). Therefore, discrepancies sometimes occurred. Since it has been reported that mutation status differs between the primary lesion and metastatic sites, we compared twelve additional somatic mutations between the primary lesion and lymph node metastatic lesion in five different genes using genomic DNA isolated from mouse no. 1 (Table S4). There were no other mutations besides L858R and T790M either in the primary lesion or in the lymph node metastasis.

### *EGFR* mutations T790M and L858R were detected in plasma in mice with metastases

**Figure 3:**
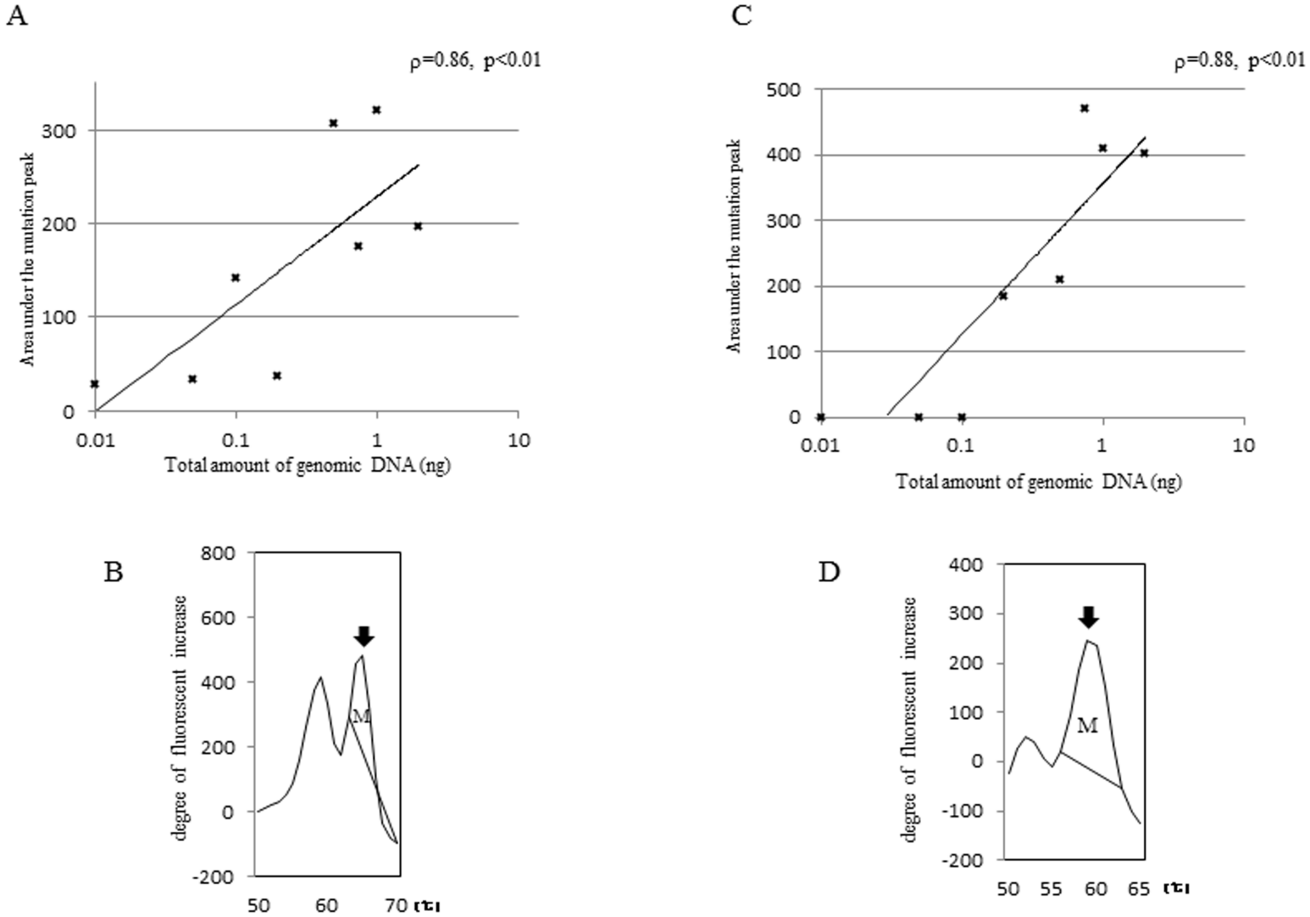
Relationship between amount of genomic DNA isolated from H1975 and areas under the mutation peaks obtained by MBP-QP. The results of T790M (A, B) and L858R (C, D) are shown. The MBP-QP method for detection of T790M (A) and L858R (C) was applied to the indicated amount of genomic DNA isolated from H1975. Black arrows indicate the peaks for T790M (B) or L858R (D) mutations. Areas under the mutation peaks (indicated as M) were defined between 63–70^ο^C for T790M, and 56–63^ο^C for L858R. These areas were calculated using the i-densy AreaAna software.

**Figure 4:**
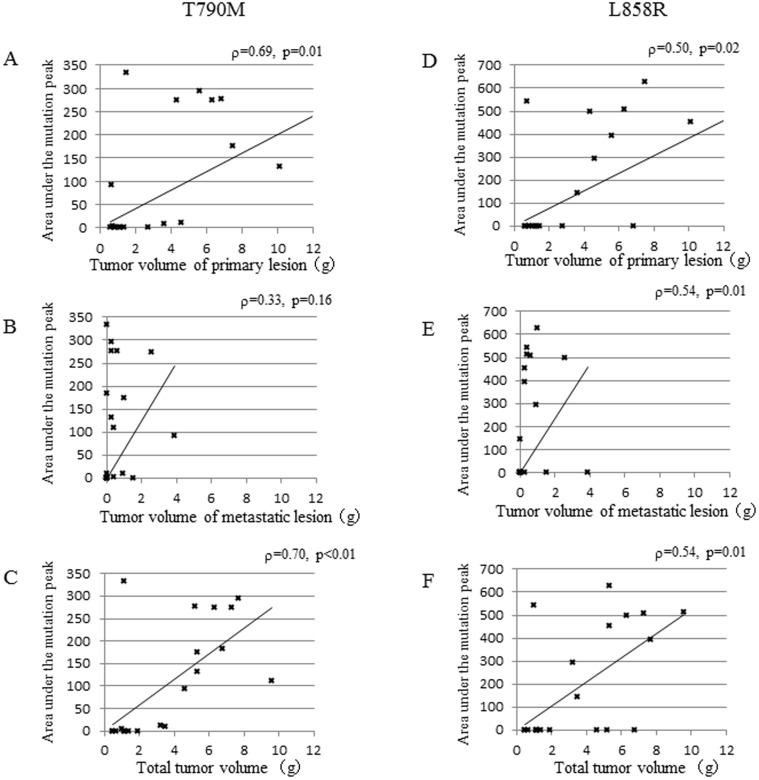
Correlation between tumor volume and area under the mutation peaks. The results of T790M (A, B, C) and L858R (D, E, F) are shown. Correlation with tumor volume of primary lesion (A, D), metastatic lesions (B, E), or total tumor lesions (C, F) and area under the mutation peaks were analyzed. After inoculation with H1975 into dorsal flanks, six mice were sacrificed monthly from one month to four months, and plasma samples were collected. T790M and L858R were determined using the MBP-QP method, and the areas under the peaks were measured using i-densy AreaAna. Tumor volumes of primary lesions and metastatic lesions were weighed after dissection. ρ: Spearman rank correlation coefficient; p: p value.

**Figure 5:**
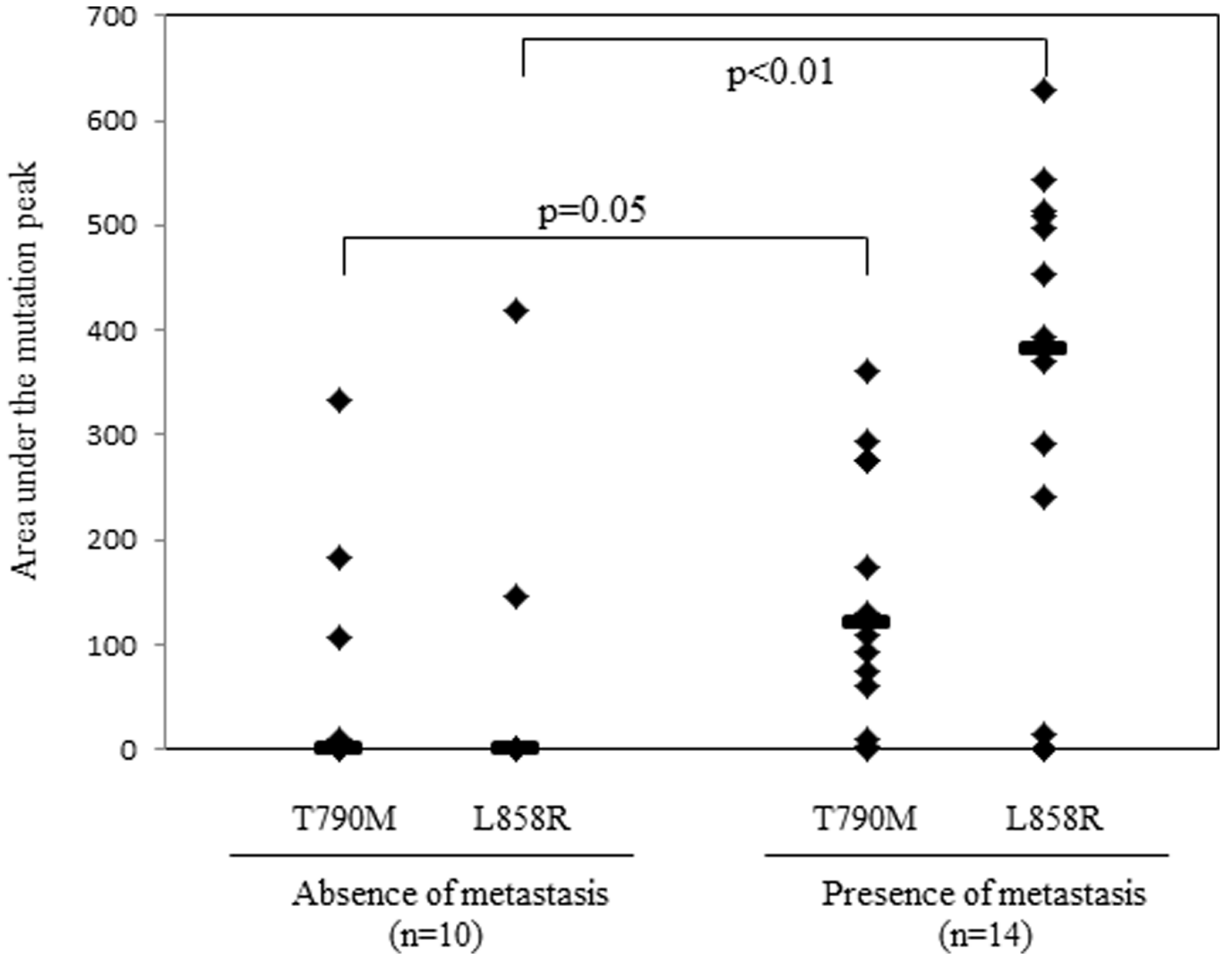
Comparison of area under the mutation peaks between absence and presence of metastasis. Among the mice examined in Figure 4, area under the mutation peaks of T790M and L858R was compared between absence and presence of metastasis. Bars; median; p: p value.

As we recently showed that T790M and L858R were detected in plasma DNA isolated from lung cancer patients using the MBP-QP method, we examined whether these mutations were detected in plasma DNA isolated from the mice. Before the experiments, we analyzed the relationship between amounts of genomic DNA isolated from H1975 and areas under the mutation peaks obtained by the MBP-QP system. The areas of both L858R and T790M were associated with amounts of mutations (Fig. 3). These results suggest that mutant allele copy number is related with area under the mutant peaks. Based on these results, we investigated the relationship between tumor progression and amount of *EGFR* mutations, T790M and/or L858R, with plasma DNA. Tumor volumes of primary and metastatic lesions, as well as *EGFR* mutation status using plasma DNA, were evaluated monthly from one to four months after inoculation with H1975 cells. The frequency of metastasis increased with time concomitant with increasing detection of *EGFR* mutations using plasma DNA (Table S5). T790M and/or L858R were detected in all mice three months after inoculation. Comparison between tumor volume and area under the mutation peak is shown in Figure 4. Tumor volume of the primary lesion was significantly correlated with area under the mutation peak (T790M; ρ = 0.69, p = 0.01, L858R; ρ = 0.50, p = 0.02), whereas that of metastatic lesions for T790M was not (ρ = 0.33, p = 0.16). Regardless of tumor volume of metastatic lesions, area under the mutation peak was relatively high. The area under the mutation peaks was significantly higher in presence of metastasis (T790M; median = 121, L858R; median = 382) compared to absence of metastasis (Fig. 5). These data suggest that the amount of mutation is correlated with tumor volume, although a large amount of free DNA is circulating in the presence of metastasis.

## Discussion

Animal models of solid tumor metastasis to date have had numerous limitations. In most models, metastasis occurred after injection into the tails of mice, or an extracellular matrix was needed for engrafting [11], [31]–[34]. Metastatic models of intravenously injected cancer cells do not reflect the first step of metastasis including cell adhesion and intravasation. So, subcutaneous injection such as with our model would be more suitable for the investigation of metastasis. Another benefit of our model is that it is possible to confirm metastasis by examining mutation status of the human genome such as *EGFR*, and molecular analysis using human genome information can be applicable. In addition, a detection system for human *EGFR* mutations with plasma DNA can be used to assess tumor progression. A xenograft model using human cancer cells requires immunodeficient mice, but it has been difficult to induce reliable metastasis after subcutaneous injection into SCID or NOD/SCID mice. Recently, advanced immunodeficient mice such as NOD/SCID *IL2rγ^null^* and NOD-Rag1^null^ have been developed [11], [13], [33], [35], [36]. However, most animal models were established for reconstitution of hematoimmuno systems or hematological malignancies. We present a xenograft model with systemic metastasis of human lung cancer cells, which occurred from one month after subcutaneous innoculation, using NOJ mice.

Tumor derived circulating plasma DNA was first identified in cancer patients in the 1970's [37]. At first, free DNA in peripheral blood was quantified with radioimmunoassay, and it has been reported that the DNA concentration was higher in cancer patients than normal controls. After establishment of molecular technology such as quantitative real-time PCR, it has been clarified that the DNA integrity significantly increased in cancer patients compared to healthy volunteers, suggesting that cancer derived free DNA originated from both apoptosis and necrosis of cancer cells, whereas free DNA from normal cells was mainly from apoptosis [38], [39]. Considering the amount of free DNA, it is difficult to contend that necrosis and apoptosis of cancer cells are the only sources of free DNA, so an alternative mechanism for DNA release from cancer cells has been considered [40], [41]. Recently, extracellular DNA has been observed on the surface of cancer cells, but not normal cells, and extracellular DNA has been shown to contribute to tumor progression such as invasion and metastasis. In addition, plasma obtained from cancer patients induced transformation and tumorigenesis mediated through uptake of cancer derived DNA [42], [43]. Considering that detection of genetic alternations with plasma DNA, so called ‘liquid biopsy”, has been widely applied, it is important to clarify the mechanisms of detection of circulating DNA and contribution to tumor progression. Our animal model enables us to examine the roles of circulating DNA on tumor progression, which will be done as further investigation. We demonstrated in this paper that tumor volume of the primary lesion was correlated with the amount of tumor derived DNA in peripheral blood, and a large amount of DNA was detected regardless of tumor volume in the presence of metastasis (Fig 5). These results suggest that the tumor derived DNA was correlated with tumor progression. However, it has not been clarified whether the phenomenon is the result of, or cause of, tumor progression.

In this paper, we established an animal model with metastasis using human lung cancer cells inoculated into the immunodeficient mouse NOJ. Our animal model evidenced that metastases one month after inoculation spread to lymph nodes, liver, and lung, as well as peritoneal dissemintation, suggesting that metastases occurred both lymphogenically and hematogenously. In addition, tumor derived plasma DNA was detected using the MBP-QP method, which has been applied to humans. The amount of tumor derived DNA was associated with primary tumor volume, whereas the amount tended to be large in the presence of metastasis regardless of the volume. This animal model should be useful to investigate further the mechanisms of circulating tumor derived DNA in peripheral blood and could be applicable to a preclinical trial for a novel anti-cancer agent targeted at metastasis.

## Supporting Information

Figure S1 **Screening of lung cancer cell lines by features of epithelial mesenchymal transition.** Twenty human lung cancer cell lines were screened through expression pattern of E-cadherin and vimentin on the basis of definition of epithelial mesenchymal transition. Western blot analysis was applied to 50 µg of whole cell lysate obtained from each cell line (A), and relative expression of E-cadherin or vimentin to actin was calculated according to the density evaluated by ImageJ software (B).
(PDF)

Figure S2 **Tumor volume of xenograft engrafted with human non-small cell lung cancer cell lines.** Tumor diameters were measured every 3 days after injection of 1×10^7^ cells of non-small cell lung cancer cell lines until reduction of body weight by 10% of original weight, or appearance of metastatic manifestations. Tumor volume was calculated as ((short axis)^2^× (long axis))/2. The results are expressed as the mean ±SD of tumor volume.
(PDF)

Table S1 **Primers for direct sequencing.**
(PDF)

Table S2 **Homology between primers, probes for T790M using MBP-QP and mouse genomic DNA.**
(PDF)

Table S3 **Homology between primers, probes for L858R using MBP-QP and mouse genomic DNA.**
(PDF)

Table S4 **Comparison of mutation status related to lung cancer between primary and metastatic lesions.**
(PDF)

Table S5 **Time-dependent change of T790M and L858R with plasma DNA related with tumor volume.**
(PDF)
