# Supplementary figures and images for: Correlation between Plasma DNA and Tumor Status in an Animal Model
Source: PLoS One. 2014 Dec 2;9(12):e111881. doi: 10.1371/journal.pone.0111881 (PMC4251827; doi:10.1371/journal.pone.0111881)

Figure S1

A

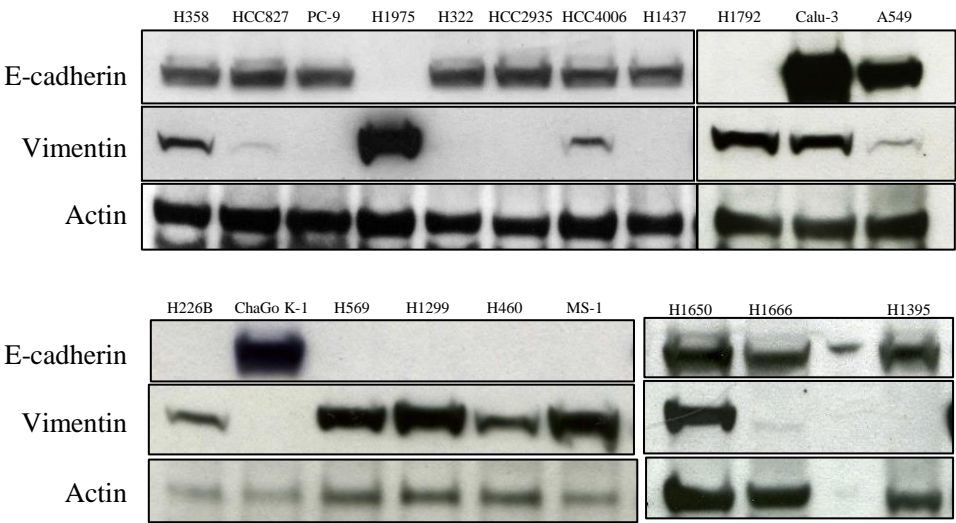

B

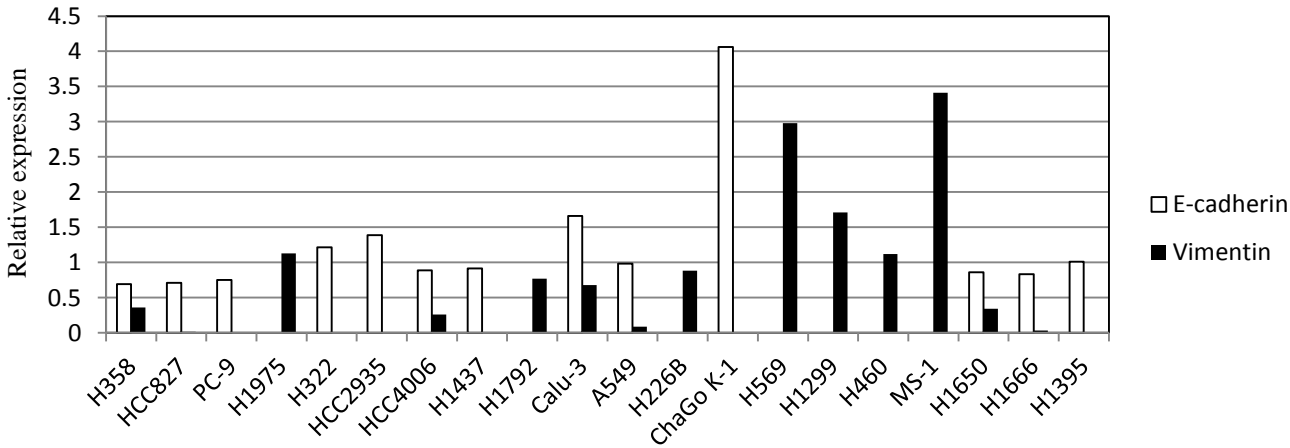

Supplement: Figure S1 — Screening of lung cancer cell lines by features of epithelial mesenchymal transition. Twenty human lung cancer cell lines were screened through expression pattern of E-cadherin and vimentin on the basis of definition of epithelial mesenchymal transition. Western blot analysis was applied to 50 µg of whole cell lysate obtained from each cell line (A), and relative expression of E-cadherin or vimentin to actin was calculated according to the density evaluated by ImageJ software (B). (PDF) [file pone.0111881.s001.pdf]

Figure S2

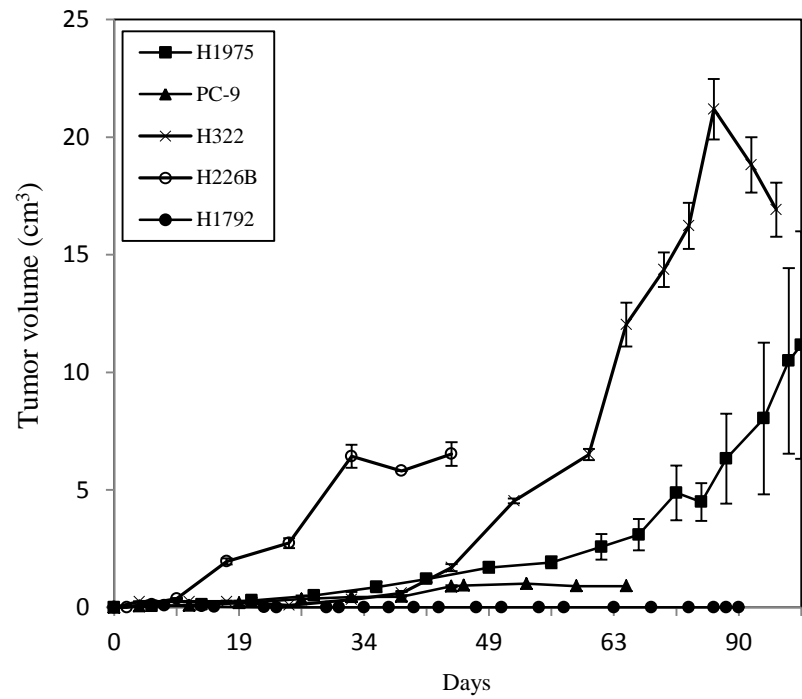

Supplement: Figure S2 — Tumor volume of xenograft engrafted with human non-small cell lung cancer cell lines. Tumor diameters were measured every 3 days after injection of 1×10^7^ cells of non-small cell lung cancer cell lines until reduction of body weight by 10% of original weight, or appearance of metastatic manifestations. Tumor volume was calculated as ((short axis)2× (long axis))/2. The results are expressed as the mean ±SD of tumor volume. (PDF) [file pone.0111881.s002.pdf]
