# Supplementary material for: Correlation between Plasma DNA and Tumor Status in an Animal Model
Source: PLoS One. 2014 Dec 2;9(12):e111881. doi: 10.1371/journal.pone.0111881 (PMC4251827; doi:10.1371/journal.pone.0111881)
Supplement: Table S1 — Primers for direct sequencing. (PDF) [file pone.0111881.s003.pdf]

Table S1. Primers for direct sequencing

AKT1-E17K	F: 5’-ACATCTGTCCTGGCACAC-3’
	R: 5’-GCCAGTGCTTGTTGCTTG-3’
BRAF-exon11	F: 5’-TCTGTTTGGCTTGACTTGACTT-3’
	R: 5’-TCACCACATTACATACTTACCATGC-3’
PIK3CA-exon9	F: 5’-GACAAAGAACAGCTCAAAGCAA-3’
	R: 5’-TTTAGCACTTACCTGTGACTCCA-3’
PIK3CA-exon20	F: 5’-GAGCAAGAGGCTTTGGAGTA-3’
	R: 5’-ATCCAATCCATTTTTGTTGTCC-3’
PTEN-exon7	F: 5’-GGTGAAGATATATTCCTCCAATTCA-3’
	R: 5’-TTCTCCCAATGAAAGTAAAGTACAAA-3’
