# Supplementary material for: Correlation between Plasma DNA and Tumor Status in an Animal Model
Source: PLoS One. 2014 Dec 2;9(12):e111881. doi: 10.1371/journal.pone.0111881 (PMC4251827; doi:10.1371/journal.pone.0111881)
Supplement: Table S2 — Homology between primers, probes for T790M using MBP-QP and mouse genomic DNA. (PDF) [file pone.0111881.s004.pdf]

Table S2. Homology between primers, probes for T790M using MBP-QP and mouse genomic DNA

<table>
<tr><th>T790M</th><th></th><th>Sequence</th><th></th><th>Homology</th></tr>
<tr><td>Primer F

mouse genomic DNA</td><td>5’-

30974</td><td>tccaggaagcctacgtgatggccag
| |||||||||| |||||||| ||
tgtaggaagcctatgtgatggctag</td><td>-3’

30998</td><td>84%</td></tr>
<tr><td>Primer R-WT

mouse genomic DNA</td><td>5’-

31160</td><td>cgcagctcatgcccttcgcgtag
| || | | | || |
caaaggtaagcacgttgggaggt</td><td>-3’

31182</td><td>39.1%</td></tr>
<tr><td>Primer R-M

mouse genomic DNA</td><td>5’-

31160</td><td>tgcagctcatgcccttcgggagca
|| | | | || || |
caaaggtaagcacgttgggaggtc</td><td>-3’

31183</td><td>41.7%</td></tr>
<tr><td>Probe

mouse genomic DNA</td><td>5’-

31150</td><td>gtgcacctcatcacgcagctca
||||| | | | ||
gtgcagattgcaaaggtaagca</td><td>-3’

31171</td><td>45.5%</td></tr>
</table>
