# Supplementary material for: Correlation between Plasma DNA and Tumor Status in an Animal Model
Source: PLoS One. 2014 Dec 2;9(12):e111881. doi: 10.1371/journal.pone.0111881 (PMC4251827; doi:10.1371/journal.pone.0111881)
Supplement: Table S3 — Homology between primers, probes for L858R using MBP-QP and mouse genomic DNA. (PDF) [file pone.0111881.s005.pdf]

Table S3. Homology between primers, probes for L858R using MBP-QP and mouse genomic DNA

<table>
<tr><th>L858R</th><th>Sequence</th><th>Homology</th></tr>
<tr><td>Primer F
mouse genomic DNA</td><td>5’- gggcatgaactacctggaagatcgg -3’
|| | | ||| || | ||
38371 aggaacgtactggtgaaaacaccgc 38395</td><td>48%</td></tr>
<tr><td>Primer R-WT
mouse genomic DNA</td><td>5’- tggccatactgctgggtagtc -3’
|||||| |||||| ||| |
38475 tggccaaactgcttggtgctg 38495</td><td>76.2%</td></tr>
<tr><td>Primer R-M
mouse genomic DNA</td><td>5’- gggctaaactgctgggtagtgt -3’
||| |||||||| ||| ||
38475 tggccaaactgcttggtgctga 38496</td><td>72.7%</td></tr>
<tr><td>Probe
mouse genomic DNA</td><td>5’- gattttgggcgggccaa -3’
||||||||| ||||| |
38464 gattttgggctggccaa 38481</td><td>94.1%</td></tr>
</table>
