# Supplementary material for: Correlation between Plasma DNA and Tumor Status in an Animal Model
Source: PLoS One. 2014 Dec 2;9(12):e111881. doi: 10.1371/journal.pone.0111881 (PMC4251827; doi:10.1371/journal.pone.0111881)
Supplement: Table S4 — Comparison of mutation status related to lung cancer between primary and metastatic lesions. (PDF) [file pone.0111881.s006.pdf]

Table S4. Comparison of mutation status related to lung cancer between primary and metastatic lesions

	EGFR L858R	*EGFR* T790M	*KRAS* codon 12/13	*AKT1* E17K	*BRAF* G466V G469A	*PIK3CA* E542K E545Q E545K	*PIK3CA* H1047R H1047L G1050D	*PTEN* R233*
H1975	+	+	WT	WT	WT	WT	WT	WT
Primary lesion	+	+	WT	WT	WT	WT	WT	WT
Lymph node metastasis	+	+	WT	WT	WT	WT	WT	WT
