# Supplementary material for: Correlation between Plasma DNA and Tumor Status in an Animal Model
Source: PLoS One. 2014 Dec 2;9(12):e111881. doi: 10.1371/journal.pone.0111881 (PMC4251827; doi:10.1371/journal.pone.0111881)
Supplement: Table S5 — Time-dependent change of T790M and L858R with plasma DNA related with tumor volume. (PDF) [file pone.0111881.s007.pdf]

Table S5. Time-dependent change of T790M and L858R with plasma DNA related with tumor volume

	No. of mouse	Dissection (days)	Tumor volume (g)			Area under mutation peak with plasma DNA	
			Primary lesion	Metastatic lesion	Total	T790M	L858R
1M	15	27	0.5	0	0.5	n.d.	n.d.
	16	27	0.5	0	0.5	n.d.	n.d.
	17	28	1.2	0	1.2	n.d.	n.d.
	18	28	1.1	0	1.1	n.d.	n.d.
	19	28	0.6	0.4	1	3.4	544
	20	32	0.4	1.5	1.9	n.d.	n.d.
2M	21	49	0.7	3.9	4.6	93	n.d.
	22	53	1.4	0	1.4	n.d.	n.d.
	23	53	2.3	0.9	3.2	11	292
	24	53	3.5	0	3.5	8.9	147
	25	53	0.7	0	0.7	n.d.	n.d.
	26	53	1.1	0	1.1	333	n.d.
3M	27	63	3.7	2.6	6.3	275	497
	28	75	6.7	0.6	7.3	275	508
	29	81	9.2	0.4	9.6	110	513
	30	81	5	0.3	5.3	131	453
	31	81	6.8	0	6.8	184	n.d.
	32	90	7.4	0.3	7.7	295	393
4M	33	94	8.1	0	8.1	106	418
	34	94	7.2	1.6	8.8	362	371
	35	97	4.3	1	5.3	175	629
	36	97	4.9	0.3	5.2	276	n.d.
	37	97	8	0.01	8.01	74	15
	38	97	7.9	0.1	8	62	240

n.d.: mutation peak was not detected.
